# Supplementary material for: Different brain networks mediate the effects of social and conditioned expectations on pain
Source: Nat Commun. 2019 Sep 10;10:4096. doi: 10.1038/s41467-019-11934-y (PMC6736972; doi:10.1038/s41467-019-11934-y)
Supplement: Supplementary file 1 — Supplementary Information [file 41467_2019_11934_MOESM1_ESM.pdf]

Supplementary Information

Different brain networks mediate the effects of social and conditioned expectations on pain

Koban et al.

Supplementary Figure 1

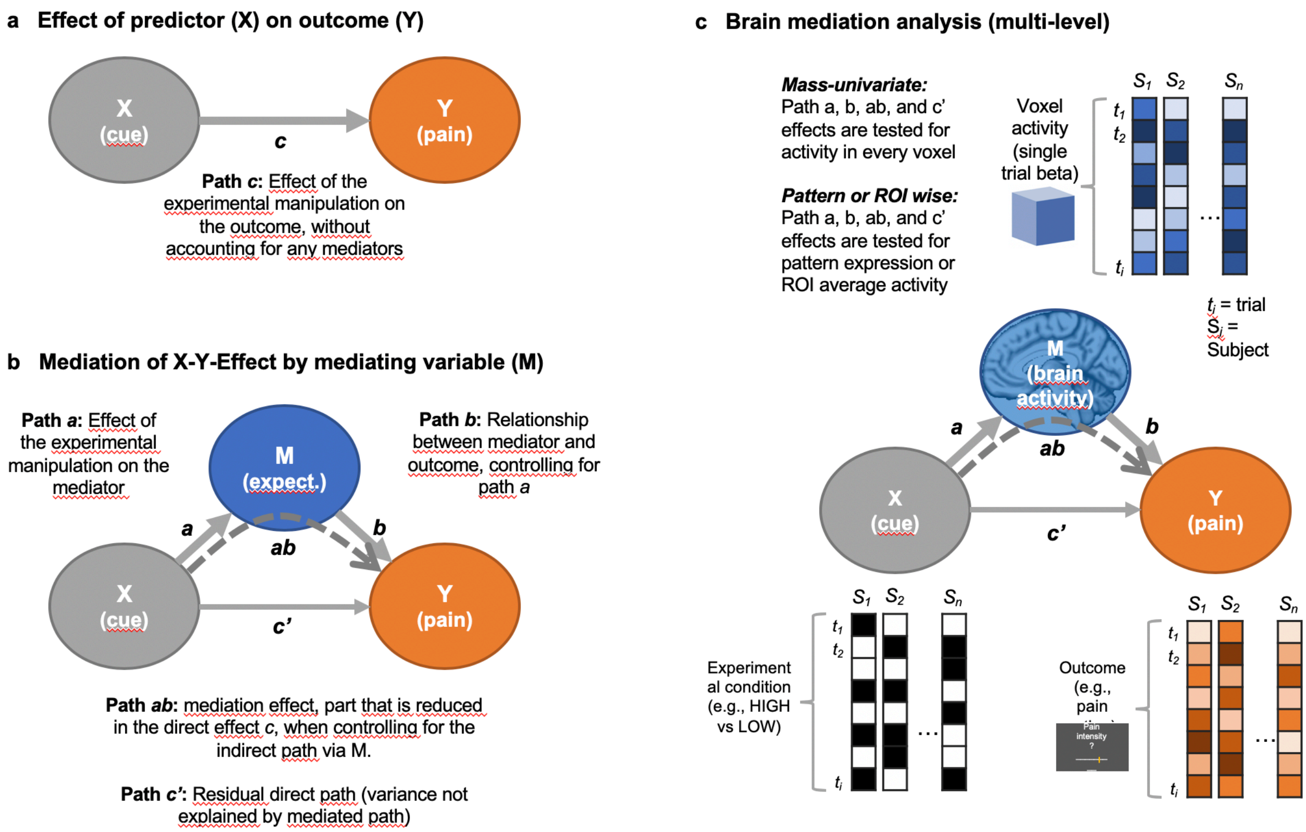

Supplementary Figure 1. A general overview of mediation analysis. A) The starting point of a mediation analysis is a directional relationship between an initial variable (X) and an outcome or dependent variable (Y). This effect is the Path *c*, the direct path from X to Y. Often, as in this case, X is an experimental manipulation, which provides stronger justification for inferring causal effects. B) Mediation tests whether a third variable (the mediator, M) can explain the effect of X on Y. Mediation assumes that X causes a change in M (Path *a*), which in turn leads to changes in Y (Path *b*, controlling for Path *a* effects). For instance, a 'HIGH' cue (compared to a LOW cue, X) may change expectations (M), which in turn change experienced pain (Y). The mediation effect (Path *ab*) is significant if it reduces the variance explained by the direct path (Path *c'* – Path *c*). C) In a multilevel brain mediation analysis, individual single-trial estimates of brain activity can be used to test for mediation effects. In a mediation effect parametric mapping analysis, the effects shown in panel B) are tested for every voxel in the brain (or in a subset of ROIs). Alternatively, multivariate pattern expression or average region of interest (ROI) activity can be tested as a mediating variable. Mediation effects are tested both on the single-person-, and across single-person- and group-level.

Supplementary Figure 2

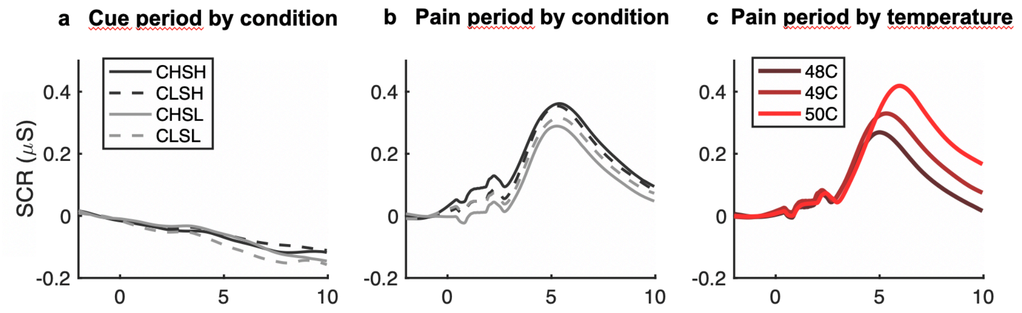

Supplementary Figure 2. Average skin conductance responses. A) SCR time-locked to cues (onset at 0s) for the four different conditions (CHSH = CS_{HIGH}/$Social_{HIGH}$, CLSH = CS_{LOW}/$Social_{HIGH}$, CHSL =CS_{HIGH}/$Social_{LOW}$, and CLSL = CS_{LOW}/$Social_{LOW}$). B) SCR time-locked to the onset of the heat pain stimulus (0 sec) by the four experimental conditions for 49°C stimuli**. C)** SCR time-locked to the onset of heat pain separately for the three different temperatures, 48°, 49°, and 50°C. Source data are provided as a Source Data file.

Supplementary Figure 3

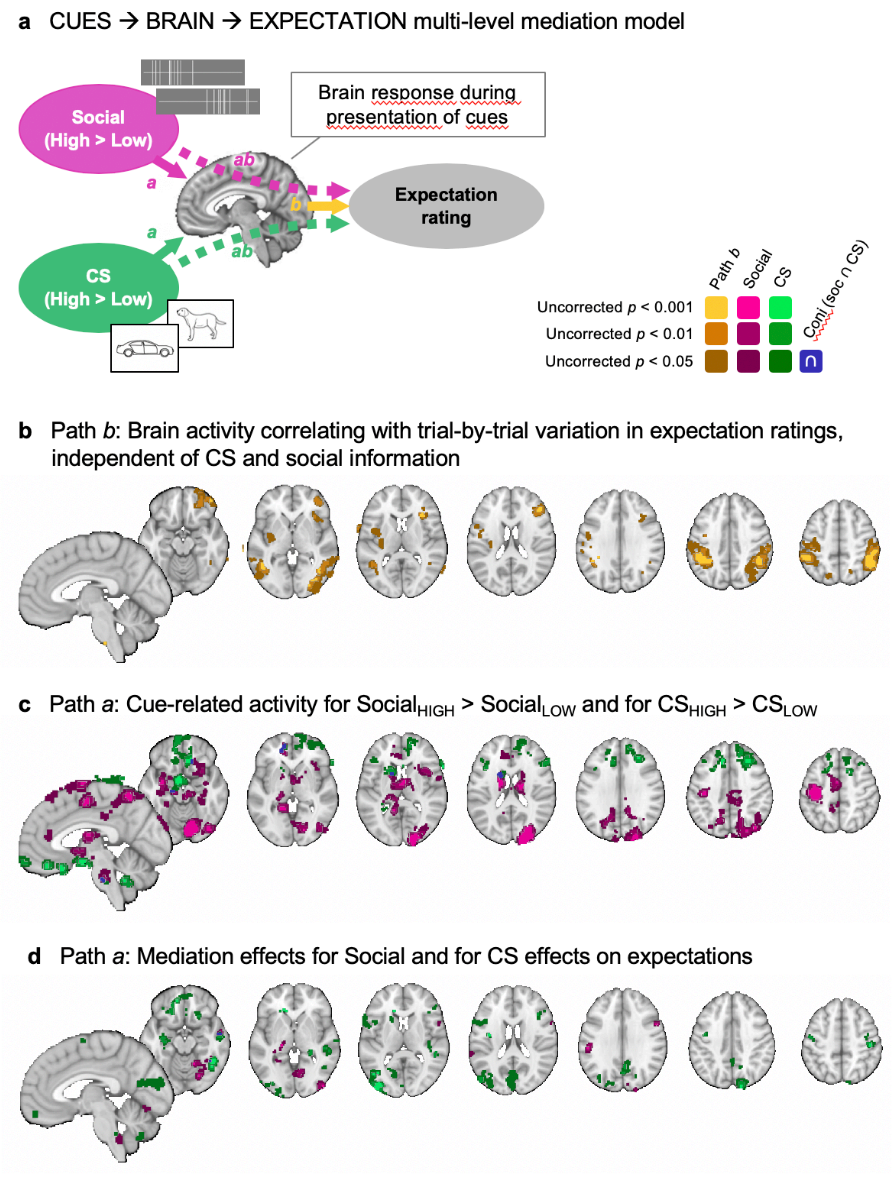

Supplementary Figure 3. Multilevel mediation results for cue-related activity. A) Two supplementary mediation models explored brain activity during the presentation of the cues, and tested which areas mediated effects of social information (in purple) and CS (in green) on expectation ratings. B) Brain activity for Path *b* effects, i.e. areas that correlated with trial-by-trial variation in expectation ratings, after controlling for social information and CS effects. C) Brain activity for Path *a* effects. In purple, activity for $\text{Social}_{\text{HIGH}}$ > $\text{Social}_{\text{LOW}}$ cues; in green activity for CS_{HIGH} > CS_{LOW} cues; conjunction (overlap) of both effects in dark blue. At uncorrected thresholds, increased activity for CS_{HIGH} compared to CS_{LOW} cues was found in vmPFC, striatum, and superior frontal gyrus, whereas increased activity for $\text{Social}_{\text{HIGH}}$ versus $\text{Social}_{\text{LOW}}$ cues was found in anterior insula, amygdala, hippocampus, thalamus, brainstem, occipital, and medial frontal and parietal areas. Small areas of overlapping social and CS Path *a* effects were observed in the vmPFC, striatum, and the brainstem. D) Brain activity formally mediating social information (in purple) and CS effects (in green) on expectation ratings. Hippocampus and medial prefrontal areas mediated CS effects on expectations, while a small cluster in the right dlPFC, in the left postcentral gyrus, and several occipital areas mediated social cue effects on expectations. Activation maps are displayed at $p < 0.001$ uncorrected, with adjacent voxels at $p < 0.01$ and $p < 0.05$ uncorrected.

Supplementary Figure 4

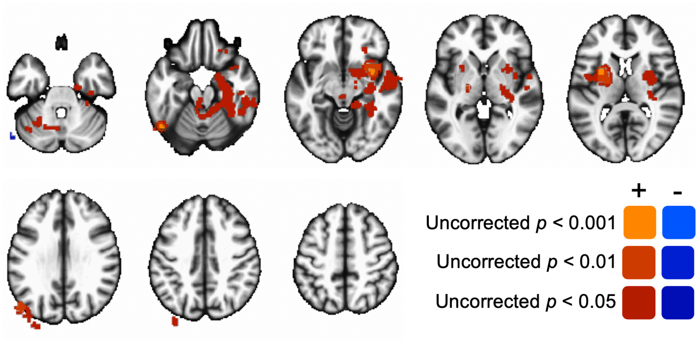

Supplementary Figure 4. Social x CS interaction effects during presentation of the cues. Several brain areas, including medial and inferior temporal lobe (including hippocampus), putamen, and ventral parts of the insula, showed increased activity for congruent compared to incongruent cue conditions ([CS_{HIGH}-$Social_{HIGH}$, CS_{LOW}-$Social_{LOW}$] > [CS_{HIGH}-$Social_{LOW}$, CS_{LOW}-$Social_{HIGH}$]), displayed in orange to red (thresholded at $p < 0.001$ *uncorrected* for multiple comparisons, with adjacent areas displayed at $p < 0.01$ and $p < 0.05$ uncorrected). Almost no areas showed the opposite effect (in blue). No meaningful interaction effects were found at the time of pain stimulation, in line with the additive effects of CS and social information on pain reports.

Supplementary Figure 5

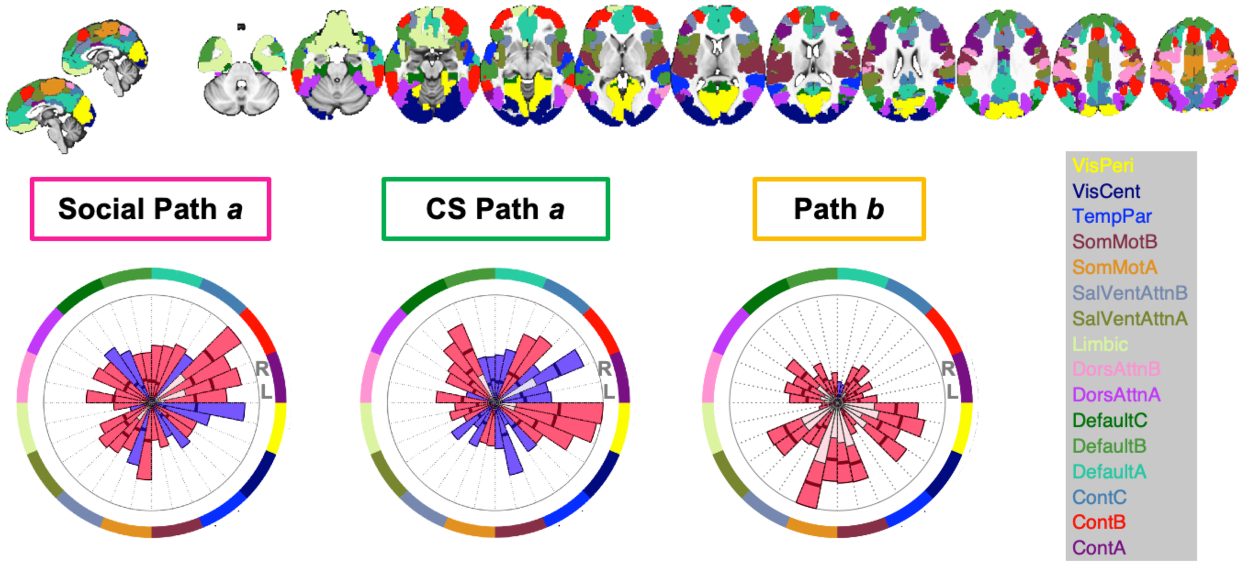

Supplementary Figure 5. Path *a* (for Social and CS) and Path *b* average beta weights in 16 bilateral (32) networks. Top row illustrates the brain parcellation used (16 bilateral subnetworks[1]). Wedge plots depict mean beta values across voxels for each parcel (R, L = right, left hemisphere). Red wedges indicate positive and blue wedges indicate negative values. Darker areas indicate SEM across individuals. Outer ring colors match the color coding of the brain display and the network names shown on the right. Source data are provided as a Source Data file.

Supplementary Figure 6

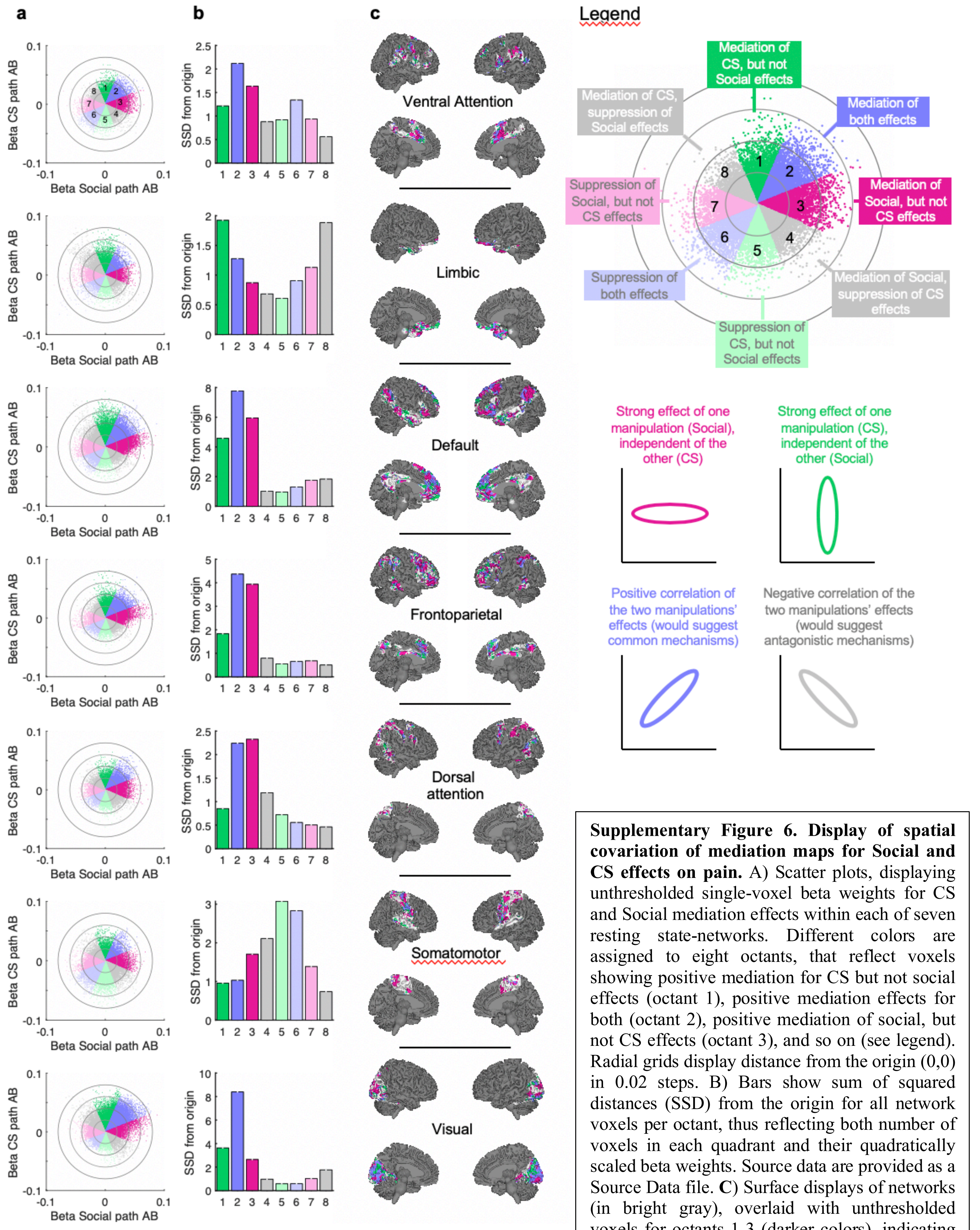

Supplementary Figure 6. Display of spatial covariation of mediation maps for Social and CS effects on pain. A) Scatter plots, displaying unthresholded single-voxel beta weights for CS and Social mediation effects within each of seven resting state-networks. Different colors are assigned to eight octants, that reflect voxels showing positive mediation for CS but not social effects (octant 1), positive mediation effects for both (octant 2), positive mediation of social, but not CS effects (octant 3), and so on (see legend). Radial grids display distance from the origin (0,0) in 0.02 steps. B) Bars show sum of squared distances (SSD) from the origin for all network voxels per octant, thus reflecting both number of voxels in each quadrant and their quadratically scaled beta weights. Source data are provided as a Source Data file. **C**) Surface displays of networks (in bright gray), overlaid with unthresholded voxels for octants 1-3 (darker colors), indicating weights positively modulated by CS (octant 1), by social information (octant 3), or by both (octant 2). Note that those maps are purely descriptive and unthresholded illustrations of mediation beta weights.

Supplementary Table 1. Path *B* effects of mediation analyses (FDR corrected $q < 0.05$), indicative of brain activity correlated with pain ratings, independent of social and CS information. Cortical regions (Ctx) are labeled based on the multimodal cortical parcellation by Glasser et al.,[2], basal ganglia regions are based on Pauli et al.[3], cerebellar regions on Diedrichsen, et al. [4], and brainstem regions based on a combination of studies[5,6]. Large-scale network names are based on the Schaefer et al.[1] resting-state parcellation. The entire combined anatomical atlas is available on Github:
https://github.com/canlab/Neuroimaging_Pattern_Masks/tree/master/Atlases_and_parcellations/2018_Wager_combined_atlas. This repository includes multiple atlases and other meta-analytic and multivariate maps. Tools for manipulating and analyzing this and other atlases are in the CANlab Core Tools repository: https://github.com/canlab/CanlabCore.

Region	Volume (mm^3)	X	Y	Z	Max(z)	Large-scale region or network
Path B positive effects (greater activity → higher pain, controlling for Path A effects)						
Putamen_Pp_L	32000	-36	-8	8	4.14	Basal_ganglia
Putamen_Pp_R	30992	42	-6	12	4.10	Basal_ganglia
Cblm_CrusII_R	80	26	-78	-50	3.57	Cerebellum
Cblm_CrusII_R	168	30	-78	-44	3.46	Cerebellum
Cblm_CrusI_R	376	48	-64	-40	3.66	Cerebellum
Cblm_CrusII_L	976	-14	-72	-36	4.08	Cerebellum
Cblm_CrusI_R	128	34	-74	-26	3.45	Cerebellum
Cblm_VI_R	120	22	-62	-16	3.26	Cerebellum
Ctx_TE2p_L	448	-48	-32	-32	4.20	Cortex_Dorsal_AttentionA
Ctx_TE2p_L	688	-38	-48	-12	3.77	Cortex_Dorsal_AttentionA
Ctx_FST_R	4464	36	-68	-6	3.89	Cortex_Dorsal_AttentionA
Ctx_FEF_L	224	-36	-12	48	3.63	Cortex_Dorsal_AttentionB
Ctx_TE1m_L	472	-66	-26	-26	4.07	Cortex_Fronto_ParietalB
Ctx_TF_R	128	44	-24	-30	3.58	Cortex_Limbic
Ctx_4_R	1784	44	-14	40	3.98	Cortex_SomatomotorA
Ctx_4_L	58368	-2	-24	60	4.64	Cortex_SomatomotorA
Ctx_55b_R	176	56	2	44	3.43	Cortex_Ventral_AttentionA
Ctx_V4_R	136	28	-84	2	3.29	Cortex_Visual_Central
Ctx_LO1_R	712	32	-82	6	3.83	Cortex_Visual_Central
Ctx_V1_L	61952	-4	-68	-6	4.27	Cortex_Visual_Peripheral
Ctx_V1_L	144	-18	-102	0	3.37	Cortex_Visual_Peripheral
Thal_VL	1864	-12	-18	0	3.83	Diencephalon
Thal_VPL	1344	14	-20	4	3.89	Diencephalon
Thal_LP	248	20	-22	14	3.60	Diencephalon
Path B negative effects (greater activity → lower pain, controlling for Path A effects)						
V_Striatum_L	672	-2	22	-4	-3.81	Basal_ganglia
Ctx_TGd_L	928	-38	24	-32	-3.48	Cortex_Default_ModeB

Supplementary Table 2. Path *A* effects of the whole brain mediation analysis for the SOCIAL information effect (FDR corrected $q < 0.05$), showing brain activity changes in response to the social information manipulation ($Social_{HIGH}$ *versus* $Social_{LOW}$), controlling for CS condition.

Region	Volume (mm³)	X	Y	Z	Max(Z)	Large-scale region or network
Path A positive effects ($Social_{HIGH}$ > $Social_{LOW}$)						
Amygdala_CM_	464	-24	-4	-10	3.88	Amygdala
GPe_R	848	18	4	-6	3.65	Basal_ganglia
Cblm_I_IV_R	480	26	-30	-34	3.78	Cerebellum
Cblm_CrusI_L	192	-36	-70	-30	3.71	Cerebellum
Cblm_VI_R	560	26	-62	-26	4.18	Cerebellum
Ctx_9a_R	448	24	64	20	4.31	Cortex_Default_ModeA
Ctx_p24_L	872	-4	30	24	3.74	Cortex_Default_ModeA
Ctx_55b_L	944	-48	6	40	4.14	Cortex_Default_ModeB
Ctx_8BL_R	1176	12	48	44	5.05	Cortex_Default_ModeB
Ctx_IP1_R	432	34	-56	40	3.94	Cortex_Dorsal_AttentionA
Ctx_LIPv_L	1280	-30	-56	48	4.15	Cortex_Dorsal_AttentionA
Ctx_7PC_R	568	28	-44	68	4.07	Cortex_Dorsal_AttentionA
Ctx_PFop_L	784	-60	-20	30	4.11	Cortex_Dorsal_AttentionB
Ctx_PFt_L	464	-44	-24	34	4.05	Cortex_Dorsal_AttentionB
Ctx_PHT_L	5808	-48	-50	-14	4.57	Cortex_Fronto_ParietalA
Ctx_p9_46v_R	224	50	26	38	3.35	Cortex_Fronto_ParietalA
Ctx_TE1p_R	9736	60	-42	-14	4.87	Cortex_Fronto_ParietalB
Ctx_a47r_L	128	-32	40	-14	3.31	Cortex_Fronto_ParietalB
Ctx_a10p_L	400	-20	58	-8	3.90	Cortex_Fronto_ParietalB
Ctx_p47r_L	408	-52	38	-2	3.79	Cortex_Fronto_ParietalB
Ctx_PFm_R	2304	48	-44	50	4.06	Cortex_Fronto_ParietalB
Ctx_TE2a_L	1752	-54	-14	-38	4.07	Cortex_Limbic
Ctx_1_R	3072	26	-28	66	4.54	Cortex_SomatomotorA
Ctx_OP1_R	384	44	-24	14	3.53	Cortex_SomatomotorB
Ctx_OP4_L	360	-56	-14	18	3.82	Cortex_SomatomotorB
Ctx_6v_R	1784	52	14	30	4.03	Cortex_SomatomotorB
Ctx_MI_L	3504	-44	12	-6	4.03	Cortex_Ventral_AttentionA
Ctx_PF_L	1752	-48	-38	44	4.13	Cortex_Ventral_AttentionA
Ctx_AVI_R	5888	40	22	-6	5.19	Cortex_Ventral_AttentionB
Ctx_p47r_R	1048	44	40	-2	3.99	Cortex_Ventral_AttentionB
Ctx_d32_R	1352	8	34	20	3.97	Cortex_Ventral_AttentionB
Ctx_33pr_R	768	2	2	30	3.86	Cortex_Ventral_AttentionB
Ctx_V4_R	1656	40	-88	-4	3.94	Cortex_Visual_Central
Ctx_V2_L	1480	-14	-82	-14	3.98	Cortex_Visual_Peripheral
Ctx_V1_L	984	-6	-88	-2	4.22	Cortex_Visual_Peripheral
Thal_VPL	760	-20	-20	10	4.36	Diencephalon
Thal_VL	984	10	-16	8	4.46	Diencephalon
No label	328	-52	48	4	3.72	
Path A negative effects ($Social_{LOW}$ > $Social_{HIGH}$)						
Bstem_Pons_L	1488	-4	-22	-42	-3.69	Brainstem
Ctx_4_L	160	-36	-26	50	-3.51	Cortex_SomatomotorA

Supplementary Table 3. Path *AB* effects of the whole brain mediation analysis for the SOCIAL information effect (FDR corrected $q < 0.05$), indicating voxels that significantly mediate the relationship between social information and pain ratings (controlling for CS condition).

Region	Volume (mm^3)	X	Y	Z	Max(z)	Large-scale region or network
Path AB positive effects (mediation of social information effect on pain)						
Amygdala_LB_	248	-12	-2	-22	5.67	Amygdala
Bstem_Ponscv_R	672	8	-20	-42	6.99	Brainstem
Bstem_Ponscd	224	8	-40	-34	3.66	Brainstem
Cblm_CrusII_R	2392	32	-72	-44	7.03	Cerebellum
Cblm_VIIIa_R	72	8	-68	-50	5.31	Cerebellum
Cblm_CrusI_R	256	58	-58	-38	4.57	Cerebellum
Cblm_X_L	144	-20	-34	-38	3.39	Cerebellum
Cblm_CrusI_R	248	44	-68	-34	5.17	Cerebellum
Cblm_CrusI_R	192	52	-70	-30	4.92	Cerebellum
Cblm_I_IV_R	288	2	-48	-22	3.84	Cerebellum
Cblm_CrusI_L	128	-38	-70	-20	3.80	Cerebellum
Cblm_VI_L	176	-22	-70	-16	3.36	Cerebellum
Ctx_s6_8_R	280	16	20	60	4.28	Cortex_Default_ModeA
Ctx_TGd_L	144	-50	8	-34	3.47	Cortex_Default_ModeB
Ctx_TGd_L	216	-62	4	-24	3.97	Cortex_Default_ModeB
Ctx_47l_L	208	-54	28	-10	4.09	Cortex_Default_ModeB
Ctx_STSvp_R	280	60	-34	-6	3.54	Cortex_Default_ModeB
Ctx_45_L	344	-54	18	2	3.59	Cortex_Default_ModeB
Ctx_9a_L	856	-18	48	26	4.08	Cortex_Default_ModeB
Ctx_9p_L	144	-16	44	34	3.48	Cortex_Default_ModeB
Ctx_9p_L	440	-14	42	44	4.10	Cortex_Default_ModeB
Ctx_PH_R	224	36	-52	-8	5.62	Cortex_Dorsal_AttentionA
Ctx_VIP_L	256	-16	-58	66	3.58	Cortex_Dorsal_AttentionA
Ctx_VIP_L	176	-18	-60	72	4.07	Cortex_Dorsal_AttentionA
Ctx_7PC_R	304	28	-52	72	4.68	Cortex_Dorsal_AttentionA
Ctx_7Am_L	320	-16	-58	56	4.03	Cortex_Dorsal_AttentionB
Ctx_6a_L	240	-18	-2	60	5.11	Cortex_Dorsal_AttentionB
Ctx_7PC_L	552	-36	-48	64	4.44	Cortex_Dorsal_AttentionB
Ctx_AIP_L	5208	-34	-50	42	6.43	Cortex_Fronto_ParietalA
Ctx_8C_L	648	-36	12	38	5.39	Cortex_Fronto_ParietalA
Ctx_6ma_L	296	-14	-6	78	5.82	Cortex_Fronto_ParietalA
Ctx_TE1m_R	1296	62	-32	-18	6.28	Cortex_Fronto_ParietalB
Ctx_TE1p_R	176	50	-44	-14	4.54	Cortex_Fronto_ParietalB
Ctx_TE1p_L	1032	-60	-36	-10	5.63	Cortex_Fronto_ParietalB
Ctx_a47r_L	752	-38	58	-8	4.37	Cortex_Fronto_ParietalB
Ctx_a10p_R	144	26	54	-8	3.53	Cortex_Fronto_ParietalB
Ctx_a10p_R	176	24	56	-4	3.75	Cortex_Fronto_ParietalB
Ctx_8BM_L	400	-10	36	32	3.94	Cortex_Fronto_ParietalB
Ctx_PFm_R	128	60	-50	28	3.53	Cortex_Fronto_ParietalB
Ctx_8Av_R	1480	46	16	38	4.93	Cortex_Fronto_ParietalB
Ctx_PFm_L	712	-48	-52	34	5.04	Cortex_Fronto_ParietalB
Ctx_PFm_L	328	-54	-62	42	3.57	Cortex_Fronto_ParietalB
Ctx_PFm_L	176	-42	-58	46	3.40	Cortex_Fronto_ParietalB

Ctx_PFm_L	368	-40	-60	54	3.54	Cortex_Fronto_ParietalB
Ctx_PeEc_R	224	16	-10	-38	3.92	Cortex_Limbic
Ctx_OFC_L	648	-4	30	-28	7.03	Cortex_Limbic
Ctx_TGd_R	384	36	18	-28	4.55	Cortex_Limbic
Ctx_TGd_R	160	50	8	-26	3.86	Cortex_Limbic
Ctx_10pp_L	144	-18	50	-20	3.40	Cortex_Limbic
Ctx_10pp_L	176	-14	70	-4	4.38	Cortex_Limbic
Ctx_4_R	280	46	-6	44	3.64	Cortex_SomatomotorA
Ctx_PoI1_L	144	-44	-8	-18	4.23	Cortex_Ventral_AttentionA
Ctx_FOP4_L	216	-26	22	14	3.84	Cortex_Ventral_AttentionA
Ctx_6r_L	248	-52	8	20	3.95	Cortex_Ventral_AttentionA
Ctx_6r_R	512	46	2	22	5.38	Cortex_Ventral_AttentionA
Ctx_PF_L	632	-66	-22	40	4.37	Cortex_Ventral_AttentionA
Ctx_11l_L	304	-26	36	-10	4.44	Cortex_Ventral_AttentionB
Ctx_11l_L	128	-28	42	-8	4.19	Cortex_Ventral_AttentionB
Ctx_a9_46v_L	176	-36	42	14	4.30	Cortex_Ventral_AttentionB
Ctx_46_R	264	30	28	34	4.33	Cortex_Ventral_AttentionB
Ctx_V2_R	696	6	-84	-4	5.11	Cortex_Visual_Central
Ctx_V2_L	1344	-8	-84	-6	4.08	Cortex_Visual_Peripheral
No label	160	28	-48	12	4.52	--
No label	192	36	-92	16	3.64	--
No label	168	40	-90	18	3.49	--
Path AB negative effects (suppression of social information effects on pain)						
Caudate_Ca_R	176	12	12	10	-3.78	Basal_ganglia
Ctx_TE2a_R	256	54	-20	-38	-3.68	Cortex_Limbic
Ctx_PF_R	256	52	-36	26	-3.50	Cortex_Ventral_AttentionA
Ctx_AVI_R	208	34	18	-2	-3.75	Cortex_Ventral_AttentionB
Ctx_ProS_L	688	-26	-60	10	-3.89	Cortex_Visual_Peripheral
Ctx_V6A_R	192	20	-86	38	-3.45	Cortex_Visual_Peripheral

Supplementary Table 4. Path *A* effects of the whole brain mediation analysis for the CS effect (FDR corrected *q* <0.05), indicating brain activity changes due to the CS manipulation (CS_{HIGH} versus CS_{LOW}), controlling for social information.

Region	Volume (mm^3)	X	Y	Z	Max(z)	Large-scale region or network
Path A positive effects (CS_{HIGH} > CS_{LOW})						
V_Striatum_L	144	-2	22	6	4.18	Basal_ganglia
V_Striatum_L	504	-12	20	12	4.16	Basal_ganglia
Cblm_VIIIa_L	368	-34	-38	-44	3.70	Cerebellum
Cblm_VI_L	928	-6	-64	-20	4.62	Cerebellum
Ctx_MIP_L	1144	-20	-66	42	4.13	Cortex_Dorsal_AttentionA
Ctx_6a_L	352	-32	-12	48	3.78	Cortex_Dorsal_AttentionB
Ctx_POS2_L	128	-20	-60	26	3.41	Cortex_Fronto_ParietalC
Ctx_TGv_R	984	30	2	-46	4.76	Cortex_Limbic
Ctx_TGv_R	352	30	-10	-44	3.71	Cortex_Limbic
Ctx_V2_L	552	-16	-76	-6	4.10	Cortex_Visual_Peripheral
Ctx_ProS_R	368	24	-56	12	3.73	Cortex_Visual_Peripheral
Ctx_ProS_R	128	20	-48	12	3.79	Cortex_Visual_Peripheral
CA1_Hippocampus_	144	-34	-14	-18	4.12	Hippocampus
Path A negative effects (CS_{LOW} > CS_{HIGH})						
Cblm_CrusII_R	544	26	-84	-42	-3.52	Cerebellum
Ctx_a47r_L	368	-34	60	4	-3.61	Cortex_Fronto_ParietalB

Supplementary Table 5. Path *AB* effects of the whole brain mediation analysis for the CS effect (FDR corrected $q < 0.05$), indicating voxels that significantly mediate the relationship between associative learning cues (CS) and pain ratings (controlling for social information condition).

Region	Volume (mm^3)	X	Y	Z	Max(z)	Large-scale region or network
Path AB positive effects (mediation of CS effects on pain)						
Caudate_Ca_R	1272	16	20	12	6.76	Basal_ganglia
Caudate_Ca_R	280	8	12	10	4.96	Basal_ganglia
Cau_R	232	10	-2	18	5.38	Basal_ganglia
Bstem_Med_R	344	2	-32	-46	3.74	Brainstem
Cblm_X_L	184	-18	-40	-46	4.99	Cerebellum
Cblm_CrusII_L	144	-24	-64	-38	3.96	Cerebellum
Cblm_VI_L	1928	-14	-66	-30	7.03	Cerebellum
Cblm_CrusII_R	288	20	-82	-34	4.53	Cerebellum
Cblm_Interposed_R	256	2	-56	-28	4.76	Cerebellum
Cblm_Interposed_L	1000	-10	-54	-26	5.42	Cerebellum
Cblm_VI_L	1184	-30	-58	-22	7.03	Cerebellum
Cblm_CrusI_R	1160	32	-68	-18	5.15	Cerebellum
Ctx_TE1a_R	992	64	-14	-10	4.47	Cortex_Default_ModeA
Ctx_9m_R	600	12	40	10	4.35	Cortex_Default_ModeA
Ctx_d32_L	496	-12	42	14	4.69	Cortex_Default_ModeA
Ctx_TE1a_L	1944	-50	-10	-22	7.03	Cortex_Default_ModeB
Ctx_TGd_L	288	-58	8	-24	4.17	Cortex_Default_ModeB
Ctx_47s_L	1176	-32	16	-22	6.33	Cortex_Default_ModeB
Ctx_45_L	1664	-42	26	0	7.03	Cortex_Default_ModeB
Ctx_9a_L	784	-20	60	20	5.16	Cortex_Default_ModeB
Ctx_9m_L	392	-10	50	24	4.98	Cortex_Default_ModeB
Ctx_8BL_L	768	-2	50	48	4.19	Cortex_Default_ModeB
Ctx_SFL_R	320	10	20	58	4.55	Cortex_Default_ModeB
Ctx_PEF_R	240	42	-2	38	3.68	Cortex_Fronto_ParietalA
Ctx_TE1p_R	408	60	-42	-16	4.06	Cortex_Fronto_ParietalB
Ctx_a10p_L	224	-22	66	-10	4.60	Cortex_Fronto_ParietalB
Ctx_PFm_L	160	-54	-58	24	5.01	Cortex_Fronto_ParietalB
Ctx_PFm_R	1056	54	-48	34	5.49	Cortex_Fronto_ParietalB
Ctx_PFm_L	1040	-56	-58	42	7.03	Cortex_Fronto_ParietalB
Ctx_8BM_L	184	-8	20	50	3.72	Cortex_Fronto_ParietalB
Ctx_TE2a_R	320	48	-8	-38	3.83	Cortex_Limbic
Ctx_TGd_R	280	34	22	-38	4.68	Cortex_Limbic
Ctx_PeEc_R	480	32	-18	-34	4.95	Cortex_Limbic
Ctx_TGd_R	144	20	16	-36	3.82	Cortex_Limbic
Ctx_TF_R	128	46	-22	-34	3.97	Cortex_Limbic
Ctx_OFC_R	624	0	38	-28	4.67	Cortex_Limbic
Ctx_Pir_L	224	-36	-4	-22	4.52	Cortex_Limbic
Ctx_TF_L	528	-46	-24	-16	5.30	Cortex_Limbic
Ctx_10pp_R	1040	2	64	-16	7.03	Cortex_Limbic
Ctx_13l_R	160	22	28	-16	4.48	Cortex_Limbic
Ctx_6v_R	736	54	14	38	5.79	Cortex_SomatomotorB
Ctx_PoI1_R	160	34	-6	-8	3.45	Cortex_Ventral_AttentionA
Ctx_6ma_R	312	14	14	70	4.90	Cortex_Ventral_AttentionA
Ctx_a32pr_R	280	10	32	16	3.62	Cortex_Ventral_AttentionB
Ctx_a9_46v_L	1032	-36	42	18	5.20	Cortex_Ventral_AttentionB

Ctx_p24_R	440	0	28	28	4.47	Cortex_Ventral_AttentionB
Ctx_V4_R	352	32	-82	-10	3.79	Cortex_Visual_Central
Ctx_PGp_L	712	-50	-78	20	5.63	Cortex_Visual_Central
Ctx_V1_R	304	20	-102	-6	4.12	Cortex_Visual_Peripheral
Ctx_ProS_L	1040	-26	-56	12	6.78	Cortex_Visual_Peripheral
Ctx_V3A_R	208	4	-86	40	4.22	Cortex_Visual_Peripheral
Thal_MD	240	-4	-18	8	4.41	Diencephalon
CA1_Hippocampus_	192	30	-6	-16	3.71	Hippocampus
CA2_Hippocampus_	576	-34	-20	-12	4.77	Hippocampus
No label	128	64	-36	-24	4.43	--
Path AB negative effects (suppression of CS effects on pain)						
Cblm_CrusI_L	160	-50	-64	-42	-3.83	Cerebellum
Cblm_CrusI_L	160	-50	-38	-40	-3.76	Cerebellum
Ctx_3b_L	512	-20	-32	68	-3.66	Cortex_SomatomotorA
Ctx_6ma_R	168	20	-8	70	-3.89	Cortex_Ventral_AttentionA

Supplementary Table 6. Spatial correlations of path contrast images with large-scale networks[7].

Network	Path *A*		Path *B*	Path *AB*	
	Social	***Cue***		***Social***	***Cue***
**Visual**	0.00	0.02	0.16	0.00	0.01
**Somatomotor**	0.01	-0.01	0.28	-0.02	-0.02
**Dorsal Attention**	0.12	0.02	-0.04	0.06	-0.01
**Ventral Attention**	0.04	-0.01	0.13	0.00	-0.01
**Limbic**	0.00	0.03	-0.06	-0.01	0.03
**Frontoparietal**	0.13	-0.01	-0.07	0.06	0.00
**Default**	0.01	-0.01	-0.09	0.03	0.06

Note. Values are Pearson's correlation coefficients for the correlation between maps, across voxels, and are intended to characterize the spatial overlap between network maps and effect maps from this study.

Supplementary Table 7. Correlations with meta-analytic term-based maps Neurosynth[8]. The data used for the analysis is available from Neurosynth at https://github.com/neurosynth/neurosynth-data/tree/master/archive (2013 release).

Path b

Term	Pearson's r
'muscle'	0.176
'foot'	0.149
'sensation'	0.138
'voluntary'	0.132
'somatosensory'	0.131
'limb'	0.126
'painrelated'	0.126
'heat'	0.125
'noxious'	0.125
'pain'	0.123

Social influence Path a		***Learning Path a***	
**Term**	**Pearson's r**	**Term**	**Pearson's r**
'counting'	0.130	'subjective'	0.159
'orthographic'	0.130	'limb'	0.155
'response'	0.121	'mentalizing'	0.150
'correct'	0.121	'physical'	0.140
'phonological'	0.117	'phonetic'	0.134
'number'	0.117	'drug'	0.130
'lexical'	0.116	'pseudowords'	0.129
'topdown'	0.114	'allele'	0.127
'distractor'	0.112	'aging'	0.127
'numerical'	0.111	'persons'	0.119

Social influence Path ab		***Learning Path ab***	
**Term**	**Pearson's r**	**Term**	**Pearson's r**
'maintenance'	0.121	'gestures'	0.140
'verbal'	0.120	'phonology'	0.120
'memory'	0.118	'syntactic'	0.118
'verb'	0.115	'sequential'	0.115
'executive'	0.114	'readers'	0.106
'preparatory'	0.114	'word'	0.101
'Attentionl'	0.113	'semantically'	0.097
'phonology'	0.106	'bimodal'	0.097
'cue'	0.105	'lexical'	0.096
'switch'	0.103	'verb'	0.096

Note. Values are Pearson's correlation coefficients and purely descriptive.

Supplementary References

1 Schaefer, A. *et al.* Local-Global Parcellation of the Human Cerebral Cortex from Intrinsic Functional Connectivity MRI. *Cereb. Cortex* **28**, 3095-3114 (2018).

2 Glasser, M. F. *et al.* A multi-modal parcellation of human cerebral cortex. *Nature* **536**, 171-178 (2016).

3 Pauli, W. M., O'Reilly, R. C., Yarkoni, T. & Wager, T. D. Regional specialization within the human striatum for diverse psychological functions. *PNAS* **113**, 1907-1912 (2016).

4 Diedrichsen, J., Balsters, J. H., Flavell, J., Cussans, E. & Ramnani, N. A probabilistic MR atlas of the human cerebellum. *NeuroImage* **46**, 39-46 (2009).

5 Shen, X., Tokoglu, F., Papademetris, X. & Constable, R. T. Groupwise whole-brain parcellation from resting-state fMRI data for network node identification. *NeuroImage* **82**, 403-415 (2013).

6 Bär, K.-J. *et al.* Functional connectivity and network analysis of midbrain and brainstem nuclei. *NeuroImage* **134**, 53-63 (2016).

7 Thomas Yeo, B. T. *et al.* The organization of the human cerebral cortex estimated by intrinsic functional connectivity. *J Neurophysiol* **106**, 1125-1165 (2011).

8 Yarkoni, T., Poldrack, R. A., Nichols, T. E., Van Essen, D. C. & Wager, T. D. Large-scale automated synthesis of human functional neuroimaging data. *Nature methods* **8**, 665-670 (2011).
