# Supplementary material for: Different brain networks mediate the effects of social and conditioned expectations on pain
Source: Nat Commun. 2019 Sep 10;10:4096. doi: 10.1038/s41467-019-11934-y (PMC6736972; doi:10.1038/s41467-019-11934-y)
Supplement: Supplementary file 3 — Reporting Summary [file 41467_2019_11934_MOESM3_ESM.pdf]

nature research

Corresponding author(s): Leonie Koban

Reporting Summary

Nature Research wishes to improve the reproducibility of the work that we publish. This form provides structure for consistency and transparency in reporting. For further information on Nature Research policies, see Authors & Referees and the Editorial Policy Checklist.

Statistical parameters

When statistical analyses are reported, confirm that the following items are present in the relevant location (e.g. figure legend, table legend, main text, or Methods section).

n/a	Confirmed	
☐	☒	The exact sample size (*n*) for each experimental group/condition, given as a discrete number and unit of measurement
☐	☒	An indication of whether measurements were taken from distinct samples or whether the same sample was measured repeatedly
☐	☒	The statistical test(s) used AND whether they are one- or two-sided<br>*Only common tests should be described solely by name; describe more complex techniques in the Methods section.*
☐	☒	A description of all covariates tested
☐	☒	A description of any assumptions or corrections, such as tests of normality and adjustment for multiple comparisons
☐	☒	A full description of the statistics including central tendency (e.g. means) or other basic estimates (e.g. regression coefficient) AND variation (e.g. standard deviation) or associated estimates of uncertainty (e.g. confidence intervals)
☐	☒	For null hypothesis testing, the test statistic (e.g. *F*, *t*, *r*) with confidence intervals, effect sizes, degrees of freedom and *P* value noted<br>*Give P values as exact values whenever suitable.*
☒	☐	For Bayesian analysis, information on the choice of priors and Markov chain Monte Carlo settings
☒	☐	For hierarchical and complex designs, identification of the appropriate level for tests and full reporting of outcomes
☐	☒	Estimates of effect sizes (e.g. Cohen's *d*, Pearson's *r*), indicating how they were calculated
☐	☒	Clearly defined error bars<br>*State explicitly what error bars represent (e.g. SD, SE, CI)*

Our web collection on statistics for biologists may be useful.

Software and code

Policy information about availability of computer code

Data collection	EPrime 2 (Professionnal) for behavioral data, Acknowledge for physiological data (skin conductance)
Data analysis	Matlab 2017b, lab toolbox and custom scripts available on github.org: https://github.com/canlab

For manuscripts utilizing custom algorithms or software that are central to the research but not yet described in published literature, software must be made available to editors/reviewers upon request. We strongly encourage code deposition in a community repository (e.g. GitHub). See the Nature Research guidelines for submitting code & software for further information.

Data

Policy information about availability of data

All manuscripts must include a data availability statement. This statement should provide the following information, where applicable:

- Accession codes, unique identifiers, or web links for publicly available datasets
- A list of figures that have associated raw data
- A description of any restrictions on data availability

Source data is presented in the source data file. Other behavioral data will be made available after publication on github: https://github.com/canlab/ and functional imaging maps will be uploaded to Neurovault.org

Field-specific reporting

Please select the best fit for your research. If you are not sure, read the appropriate sections before making your selection.

☐ Life sciences ☒ Behavioural & social sciences ☐ Ecological, evolutionary & environmental sciences

For a reference copy of the document with all sections, see nature.com/authors/policies/ReportingSummary-flat.pdf

Behavioural & social sciences study design

All studies must disclose on these points even when the disclosure is negative.

Study description	quantitative study, measuring self-report pain and fMRI-BOLD responses to heat pain stimulation
Research sample	36 participants (20 female, 16 male, mean age = 27.1 years, range from 18-50) from the broader University of Colorado community and Boulder area, recruited via flyers on campus and downtown Boulder and local/university online bulletin boards. Two additional participants were excluded due to large movement artifacts and delays in the task before data analysis. All provided informed consent and were paid for their time.
Sampling strategy	Convenience sample. Sample size was determined based on two factors: 1) power considerations: based on a behavioral pilot study (Koban & Wager, 2016, Emotion), we expected a large effect size for the social and learning effects on behavioral pain reports. Based on other previous studies in our lab on pain modulation, we expected moderate-to-large effect (Cohen's d= 0.7-1) in brain responses. 30 participants (which is also the default and budgeted sample size for fMRI studies in our lab after accounting for attrition), were calculated to yield 80% power to detect effects of 0.78 and larger at P< 0.001. 2) Number of counterbalancing conditions: As we had 18 different stimulus pairings for the conditioned stimuli during this learning task, we chose 36 as a multiple of 18 (so we had an equal number of participants per stimulus-pairing).
Data collection	Data collection was conducted in the MRI scanner at the University of Colorado Boulder. Participants were alone in the scanner and used a trackball to provide ratings on a visual analog scale. All stimuli materials (visual cues and thermal pain) were controlled using Eprime software. A Medoc Pathway device with a fMRI compatible CHEPS thermode were used to deliver mildly to moderately painful heat stimuli (1s plateau duration) to the right calf of the participants.
Timing	April-December 2014
Data exclusions	Two additional participants were excluded due to delays in the experiment and large movement artifacts (based on pre-established exclusion criteria) before data analysis.
Non-participation	No participants dropped out.
Randomization	We used a within-design for all conditions of interest. Stimulus materials (which CS was high vs. low) and order of conditions was counterbalanced across and randomized within participants

Reporting for specific materials, systems and methods

Materials & experimental systems

n/a	Involved in the study
☒	☐ Unique biological materials
☒	☐ Antibodies
☒	☐ Eukaryotic cell lines
☒	☐ Palaeontology
☒	☐ Animals and other organisms
☐	☒ Human research participants

Methods

n/a	Involved in the study
☒	☐ ChIP-seq
☒	☐ Flow cytometry
☐	☒ MRI-based neuroimaging

Human research participants

Policy information about studies involving human research participants

Population characteristics	see above
Recruitment	see above

Magnetic resonance imaging

Experimental design

Design type	event-related
Design specifications	96 trials per subject, thereof 48 at medium temperature (which contains all experimental conditions of interest), split into 6 functional imaging runs
Behavioral performance measures	mean and standard deviation of pain ratings

Acquisition

Imaging type(s)	functional
Field strength	3T
Sequence & imaging parameters	26 interleaved transversal slices (3.4mm isotropic voxels); T2* weighted EPI GRAPPA sequence (TR = 1.3, TE = 25ms, flip angle = 50°, FOV = 220mm).
Area of acquisition	whole brain scan in 26 interleaved slices

Diffusion MRI ☐ Used ☒ Not used

Preprocessing

Preprocessing software	SPM8
Normalization	T1 structural MPRAGE images (1mm isomorphic voxels) were first coregistered to the mean functional image and then normalized to the SPM template using unified segmentation.
Normalization template	SPM8 MNI template
Noise and artifact removal	SPM8 was used for estimation of motion parameters. Movement regressors, their derivatives, and spikes were used in 1st level models to correct for movement artefacts.
Volume censoring	no volume censoring

Statistical modeling & inference

Model type and settings	multilevel GLM for behavioral and physiological (SCR) effects; mass univariate brain multilevel mediation model (code available on github) for brain analysis; multivariate SVM to separate learning versus conditioning effects as additional model
Effect(s) tested	Behavioral: Social-High > Social-Low on pain expectations and rating; CShigh>CSlow on pain expectation and rating;<br>Interactions with Time (number of trials) to test for learning;<br>Skin conductance: Social-High > Social-Low on SCR single trial estimates of skin conductance responses (SCR beta weights); CShigh>CSlow on SCR beta weights;<br>fMRI/BOLD activity: Mediation Path A: SocialHigh > SocialLow; CShigh > CSlow; Mediation Path B: brain activity related to pain ratings when controlling for Path A; Path AB: mediation effect of SocialHigh>SocialLow on pain ratings and of CShigh>CSlow on pain rating;<br>multivariate SVM: brain activity separating activity for SocialHigh>SocialLow versus CShigh>CSLow<br>Supplementary analysis:<br>fMRI/BOLD activity: Mediation Path A: SocialHigh > SocialLow; CShigh > CSlow during cues presentation; Mediation Path B: brain activity evoked by presentation of the cues associated with expectation ratings (controlling for Path A effects); Path AB: mediation effect of SocialHigh>SocialLow on expectation ratings and of CShigh>CSlow on expectation ratings;

Specify type of analysis: ☐ Whole brain ☐ ROI-based ☒ Both

Anatomical location(s)	main analysis were whole brain; SVM was trained and tested in union mask of univariate effects to test the hypothesis that social and CS effects were separable;
Statistic type for inference (See Eklund et al. 2016)	voxel-wise
Correction	FDR correction for multiple comparisons across the whole brain and across all three mediation paths (see Atlas, et al., 2010; Koban, et al., 2017). Some supplementary analysis (as requested during the revision) are presented at uncorrected thresholds.

Models & analysis

n/a | Involved in the study

- [x] n/a — [ ] Functional and/or effective connectivity
- [x] n/a — [ ] Graph analysis
- [ ] n/a — [x] Multivariate modeling or predictive analysis

Multivariate modeling and predictive analysis	Social vs CS effect in union of univariate effects; default regularization settings of spider toolbox and CANLAB tools, leave-one-subject out cross-validation;
